# Functional Consequences of Complementarity-determining Region Deactivation in a Multifunctional Anti-nucleic Acid Antibody*

**DOI:** 10.1074/jbc.M113.508499

**Published:** 2013-10-23

**Authors:** Jiyeon Lee, Hye-Jin Kim, Jooho Roh, Youngsil Seo, Minjae Kim, Hye-Ryeong Jun, Chuong D. Pham, Myung-Hee Kwon

**Affiliations:** From the ‡ Department of Biomedical Sciences, Graduate School, Ajou University, San 5, Woncheon-dong, Yeongtong-gu, Suwon 443-749, South Korea and; § Department of Microbiology, Ajou University School of Medicine, San 5, Woncheon-dong, Yeongtong-gu, Suwon 443-749, South Korea

**Keywords:** Antibodies, Antibody Engineering, Autoimmunity, Immunology, Molecular Biology, Anti-DNA Antibody, CDR Deactivation, Cell Penetration, DNA Hydrolysis, Multifunctional Antibody

## Abstract

Many murine monoclonal anti-DNA antibodies (Abs) derived from mice models for systemic lupus erythematosus have additional cell-penetration and/or nucleic acid-hydrolysis properties. Here, we examined the influence of deactivating each complementarity-determining region (CDR) within a multifunctional anti-nucleic acid antibody (Ab) that possesses these activities, the catalytic 3D8 single chain variable fragment (scFv). CDR-deactivated 3D8 scFv variants were generated by replacing all of the amino acids within each CDR with Gly/Ser residues. The structure of 3D8 scFv accommodated single complete CDR deactivations. Different functional activities of 3D8 scFv were affected differently depending on which CDR was deactivated. The only exception was CDR1, located within the light chain (LCDR1); deactivation of LCDR1 abolished all of the functional activities of 3D8 scFv. A hybrid Ab, HW6/3D8L1, in which the LCDR1 from an unrelated Ab (HW6) was replaced with the LCDR1 from 3D8, acquired all activities associated with the 3D8 scFv. These results suggest that the activity of a multifunctional 3D8 scFv Ab can be modulated by single complete CDR deactivation and that the LCDR1 plays a crucial role in maintaining Ab properties. This study presents a new approach for determining the role of individual CDRs in multifunctional Abs with important implications for the future of Ab engineering.

## Teaser

**Background:** A subset of anti-DNA antibodies have DNA-hydrolysis and/or cell-penetration activity.

**Results:** Deactivation of different complementarity-determining regions (CDRs) in a multifunctional anti-nucleic acid antibody alters the properties of the antibody.

**Conclusion:** CDR1 of the light chain plays a crucial role in maintaining the properties of 3D8 scFv.

**Significance:** Single complete CDR deactivation allows exploration of the molecular features of multifunctional anti-DNA antibodies.

## Introduction

Autoantibodies against DNA play a major role in the pathogenesis of the autoimmune disease systemic lupus erythematosus (1, 2). Monoclonal antibodies (mAb) against DNA isolated from murine lupus models, such as MRL-*lpr/lpr* mice and (NZB/NZW)F_1_ mice, and from systemic lupus erythematosus patients have a high frequency of positively charged residues in their complementarity-determining regions (CDRs).^2^ These basic residues contribute to high affinity DNA binding (3–5). Apart from their DNA binding properties, many anti-DNA mAbs derived from mice and humans have additional activities, such as cell-penetration (6–11), DNA hydrolysis and/or RNA-hydrolysis (12), or both (13, 14). However, despite accumulating evidence for the multifunctional properties of anti-DNA mAbs, few studies have addressed their structural and functional features with respect to cell-penetration (7) or DNA hydrolysis (15).

3D8 single chain variable fragment (scFv) is a murine catalytic anti-nucleic acid Ab that has nucleic acid (dsDNA, ssDNA, and RNA) binding and hydrolysis properties and cell-penetrating activity (14, 15). We reported previously the results of a mutational analysis based on the x-ray crystallographic structure of 3D8 scFv, which showed that the His residues in CDR1 of the heavy chain (HCDR1) and CDR3 of the light chain (LCDR3) are critical for DNA hydrolysis (15). 3D8 scFv is endocytosed via the caveolae-mediated pathway and localizes to the cytosol without translocating to the nucleus (14, 16). Moreover, we found that the activities of 3D8 scFv are relatively tolerant of alteration of the CDR. That is, replacing the entire HCDR3 sequence with a Tat_48–60_ peptide sequence did not impair the DNA binding, DNA-hydrolyzing, or cell-penetrating activities of 3D8 scFv (17). This indication that the CDRs of 3D8 scFv are capable of accommodating multiple sequence changes together with reports that peptides derived from the CDRs of other Abs maintain functions similar to the original Ab (10, 18–20) prompted us to study the role of each CDR in the DNA binding, DNA-hydrolyzing, and cell-penetrating properties of 3D8 scFv through a complete CDR-scaled change (single complete CDR-deactivation) rather than the more commonly employed point mutation analysis of individual CDR residues.

The purpose of this study was to investigate the role of each CDR in the activities of 3D8 scFv by analyzing the effect of single complete CDR deactivations. To do this we generated six CDR-deactivated 3D8 scFv variants, each of which contained one CDR in which all the amino acid residues were replaced with a non-charged flexible sequence of repeated Gly/Ser residues, and examined both their secondary structures and their DNA binding, DNA-hydrolysis, and cell-penetration activities. Moreover, to clarify the role of LCDR1 in 3D8 activities, we performed biochemical examination of the peptides corresponding to the 3D8 CDRs as well as a hybrid Ab, HW6/3D8L1, in which the LCDR1 of human HW6 (an anti-death receptor 5 (DR5) Ab) was replaced with the LCDR1 from 3D8.

## EXPERIMENTAL PROCEDURES

### 

#### 

##### ScFv Proteins and Peptides

CDR-replaced VH (variable domain of heavy chain) and VL (variable domain of light chain) genes were synthesized by GenScript Inc. The genes were subcloned into the pIg20 vector, which contains a Protein A tag (7 kDa), using XmaI and NcoI restriction sites. This resulted in the pIg20-scFv expression vector in which VH and VL are connected by a (Gly-4/Ser-1)_3_ linker. ScFv proteins were expressed in bacteria and purified from bacterial culture supernatants in a soluble form by IgG-Sepharose chromatography (15). The concentrations of scFv proteins (mg ml^−1^ cm^−1^) were determined using extinction coefficients at 280 nm, which were calculated from the respective amino acid sequences. Peptides N-terminally labeled with either FITC or biotin were synthesized by Peptron Inc. (Korea).

##### Circular Dichroism (CD) Spectroscopy

The far-UV CD spectra of scFv proteins were recorded on a J-20 spectropolarimeter (Jasco Inc., Japan) at a concentration of 0.5–1.0 mg/ml in Tris buffer (10 mm Tris-HCl, 10 mm NaCl, pH 7.4) at 25 °C using a thermostated cuvette with a 0.1-cm path length quartz cell. Spectra were scanned from 260 to 190 nm at a scan speed of 5 nm/min.

##### Surface Plasmon Resonance (SPR)

Kinetic measurement of the interaction between scFvs and single-stranded ssDNA was performed using a Biacore 2000 instrument (GE Healthcare ) at 25 °C. scFvs were diluted in HBS-EP (10 mm HEPES, pH 7.4, 150 mm NaCl, 3.4 mm EDTA) containing 0.005% surfactant P-20. The same buffer was used as the running buffer. The substrate, ss-(dN)_40_ DNA labeled with biotin at the 5′-end (bio-ss-(dN)_40_, 5′-CCATGAGTGATAACACTGCGGCCAACTTACTTCTGACAAC-3′), was synthesized by Integrated DNA Technologies Inc. Briefly, ss-(dN)_40_ DNA (1 μm) was immobilized on a streptavidin-coated sensor chip SA (Amersham Biosciences) at a level of 200–800 response units. 3D8-Hi proteins (serially diluted from 25 to 0.8 nm) and 3D8-Li proteins (serially diluted from 200 to 0.4 nm) were injected into the flow cell for 3 min at a flow rate of 30 μl/min. All kinetic parameters were calculated by nonlinear regression analysis according to a 1:1 binding model using BIA evaluation software (Version 3.2). The dissociation constant, *K_D_*,was calculated using the formula *K_D_* = *k*_off_/*k*_on_, where *k*_off_ and *k*_on_ are the dissociation and association rate constants, respectively.

##### Flow Cytometry

To detect internalized scFvs, HeLa cells were seeded in 6-well plates at a density of 5 × 10^5^ cells/well and incubated with 5 μm scFvs in serum-free medium for 6 h at 37 °C. The cells were washed 3 times with ice-cold PBS and fixed with 4% paraformaldehyde-PBS for 10 min at 4 °C. After washing with PBS, cell membranes were permeabilized with “P” buffer (1% BSA, 0.1% saponin, and 0.1% sodium azide in PBS) and incubated with rabbit IgG (2 μg/ml) followed by FITC-anti-rabbit IgG. Each incubation step was performed for 1 h at 4 °C followed by washing with ice-cold PBS. Finally, the cells were suspended in 4% paraformaldehyde-PBS and analyzed using a FACSCanto II flow cytometer (BD Biosciences). In some cases the permeabilization step was omitted. To detect internalized peptides, HeLa cells were incubated with 10 μm FITC-labeled peptides in serum-free TOM^TM^ medium for 2 h at 37 °C. The cells were then fixed and analyzed by flow cytometry.

##### Confocal Microscopy

To detect scFvs, HeLa cells were seeded on glass coverslips in 24-well plates at a density of 4 × 10^4^ cells/well and incubated with 5 μm scFvs in serum-free medium for 6 h at 37 °C. Cells were washed, fixed, and permeabilized as described for flow cytometry experiments. To detect peptides, HeLa cells were incubated with 10 μm FITC-labeled peptides in serum-free medium for 2 h at 37 °C and then fixed. The cell nuclei were stained with Hoechst 33342 (Vector Laboratories) for 30 min at room temperature. Images were obtained using a laser scanning confocal fluorescence microscope (model LSM710, Carl Zeiss).

##### Enzyme-linked Immunosorbent Assay (ELISA)

To assay the heparin binding activity of scFvs, various concentrations (1.3–10 μg/ml) of scFvs were incubated in 96-well polystyrene plates coated with heparin (10 μg/ml; Sigma). To assay DNA binding activity, scFvs were incubated in wells coated with bio-ss-(dN)_40_ DNA (1 μm). Bound proteins were detected using rabbit IgG (Sigma) followed by alkaline phosphatase-conjugated anti-rabbit IgG (Pierce) as described previously (15). To assay the binding of peptides to heparin or DNA, biotin-labeled peptides were incubated in wells coated with pUC57 plasmid DNA (2 μg/ml) or heparin (10 μg/ml). Bound peptides were detected using alkaline phosphatase-conjugated streptavidin (Invitrogen) and *p*-nitrophenyl phosphate substrate (Sigma). To assay the human DR5 binding activity of scFvs, wells were coated with DR5 protein (2 μg/ml) obtained from PeproTech.

##### Fluorescence Resonance Energy Transfer (FRET)-based DNA Cleavage Assay

A DNA substrate composed of 21 nucleotides labeled with 6-carboxyfluorescein (FAM) at the 5′ terminus and a black hole quencher (BHQ) at the 3′ terminus (5′ FAM-CGATGAGTGCCATGGATATAC-BHQ 3′) was generated by M-Biotech. scFvs (1 μm), peptide (1 μm), or DNase I (1 unit) was either preincubated or not with heparin (a competitor molecule; 10 μg/ml) for 10 min at room temperature before being loaded into the wells of a 96-black-well plate containing DNA substrate (250 nm). Immediately after addition to the DNA substrate, the fluorescence intensity was read in real time over a period of 6 h at 37 °C in a fluorescence detector (Molecular Devices). Each reaction was brought to a final volume of 100 μl with TBSM buffer (100 mm Tris-HCl, pH 7.4, 150 mm NaCl, 2 mm MgCl_2_).

## RESULTS

### 

#### 

##### Production of scFv Variants

**FIGURE 1.**
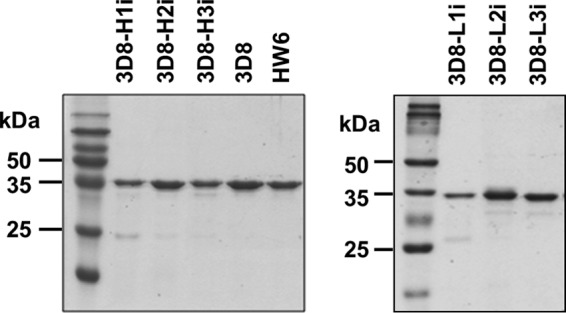
**Production of scFv Abs.** SDS-PAGE gels showing the purified 3D8 scFvs. The scFvs (10 μg) were separated on 12% SDS-PAGE gels and visualized by staining with Coomassie Blue.

ScFv proteins were purified to homogeneity as soluble proteins with molecular masses of ∼34 kDa (Fig. 1). Approximately 1–5 mg of each scFv protein was obtained from 1 liter of the supernatant of cultured *Escherichia coli*. For CDR substitution, we chose a sequence of repetitive non-charged small (Gly/Ser) residues that are commonly used for the inactivation of specific domains (21). Each scFv was named according to the position of the CDR replaced with Gly/Ser residues. For example, 3D8-H1i denotes an scFv in which HCDR1 of 3D8 was replaced, and 3D8-L3i denotes an scFv in which LCDR3 was replaced.

##### Secondary Structure Analysis of scFv Variants

**FIGURE 2.**
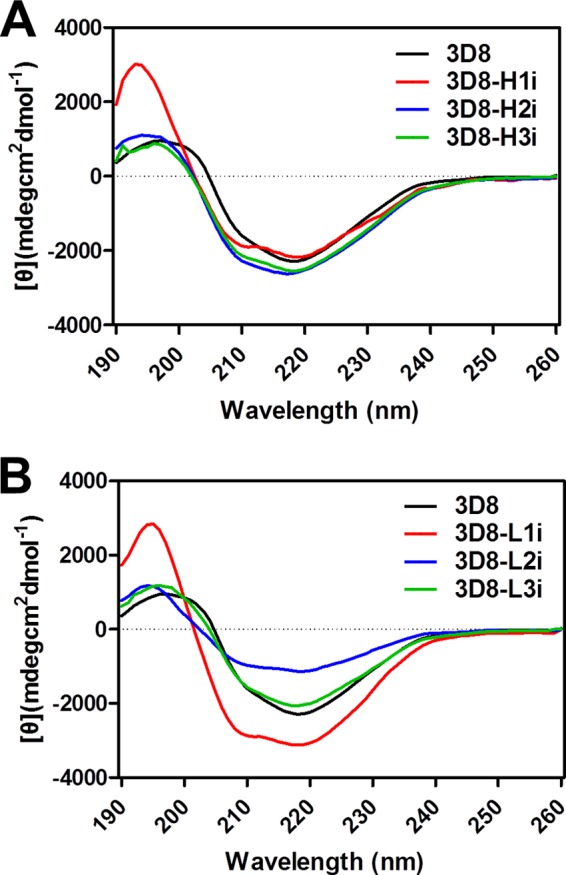
**Characterization of the secondary structures of 3D8 scFv variants by far-UV CD spectroscopy.** The far-UV CD spectra of scFv proteins were recorded in a wavelength range from 190 to 250 nm (*x* axis) and are expressed as the [θ] value, which represents the mean residue ellipticity (*y* axis). *A*, spectra of 3D8-Hi series. *B*, spectra of 3D8-Li series.

Because the deactivation of any CDR may cause improper folding of scFv, the secondary structures of the six purified scFv variants were monitored by far-UV CD spectroscopy. The far-UV CD spectra of the three 3D8-Hi (Fig. 2*A*) and three 3D8-Li variants (Fig. 2*B*) showed that their secondary structures were predominantly β-sheets, as expected for typical members of the functional immunoglobulin family, including scFvs (22, 23). These data indicate that all scFv variants folded properly and that single CDR deactivations in 3D8 scFv did not cause significant structural distortions.

##### DNA Binding Activity of scFv Variants

We first measured the affinity of the CDR-deactivated scFvs for a synthetic ss-(dN)_40_ DNA using SPR (Table 1). Wild-type 3D8 scFv bound to ss-(dN)_40_ in a concentration-dependent manner, with an affinity similar to that reported previously (15). From the kinetic binding parameters, *k*_on_ and *k*_off_, we calculated the *K_D_* of wild-type 3D8 scFv to be ∼41 nm. The binding affinities of the 3D8-Hi variants were either slightly higher (2-fold higher for 3D8-H2i) or lower (2–5-fold lower for 3D8-H1i and 3D8-H3i). With respect to the 3D8-Li variants, the affinities of 3D8-L2i and 3D8-L3i were either slightly higher (4-fold) or lower (3-fold) than that of the wild-type scFv. However, 3D8-L1i did not bind to ss-(dN)_40_. BSA (100 μm; negative control) did not bind to the surface of the ss-(dN)_40_-immobilized chip (data not shown). Taken together, these data indicate that the DNA binding activity of 3D8 is not completely abolished by the deactivation of a single CDR, except in the case of the LCDR1. This suggests that LCDR1 may be crucial for the DNA binding activity and/or structural stability of scFv.

**TABLE 1:** **SPR-derived kinetic binding parameters of the interaction between 3D8 variants and single-stranded DNA substrates** The *K_D_* values were calculated by analyzing at least five data sets using different protein concentrations.

Protein	*K_on_*	*K*_off_	*K*D
	*m*^−*1*^ *s*^−*1*^	*s*^−*1*^	*m*
3D8	(5.77 ± 0.17) × 10^4^	(2.37 ± 0.05) × 10^−3^	(4.11 ± 0.20) × 10^−8^
3D8-H1i	(1.79 ± 0.00) × 10^3^	(3.49 ± 0.13) × 10^−4^	(1.95 ± 0.07) × 10^−7^
3D8-H2i	(2.83 ± 0.07) × 10^5^	(4.75 ± 0.23) × 10^−3^	(1.68 ± 0.12) × 10^−8^
3D8-H3i	(4.50 ± 0.11) × 10^4^	(4.64 ± 0.06) × 10^−3^	(1.03 ± 0.04) × 10^−7^
3D8-L1i	(1.09 ± 0.02) × 10^2^	(1.14 ± 0.02) × 10^−2^	(1.05 ± 0.03) × 10^−4^
3D8-L2i	(1.82 ± 0.09) × 10^4^	(2.43 ± 0.09) × 10^−3^	(1.34 ± 0.13) × 10^−7^
3D8-L3i	(6.44 ± 0.20) × 10^5^	(7.23 ± 0.30) × 10^−3^	(1.13 ± 0.17) × 10^−8^

##### Cell Penetration and Heparin Binding of the scFvs

**FIGURE 3.**
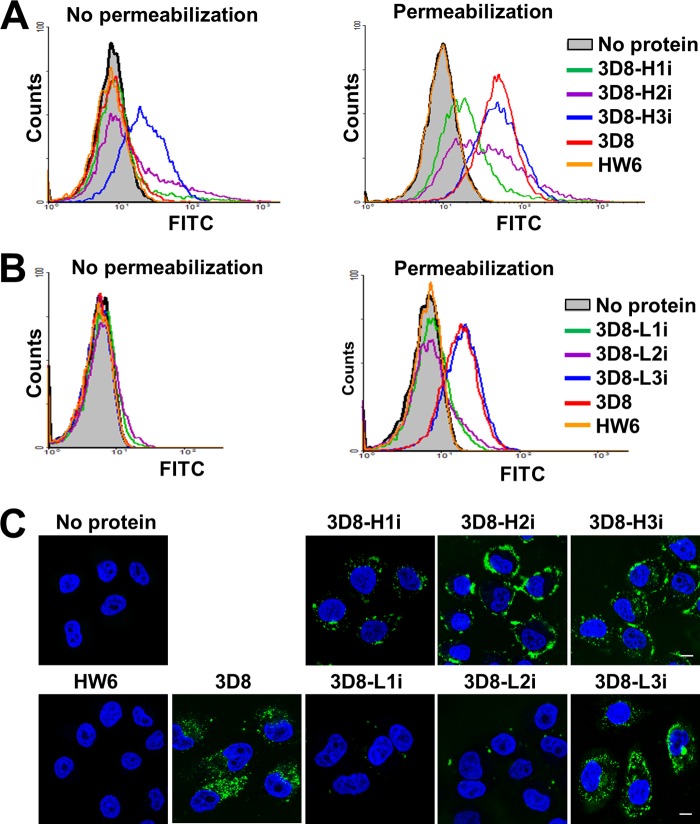
**The cell-penetrating activity of 3D8 scFv variants.** *A–C*, assays of cell-penetrating activity. HeLa cells were incubated with the 3D8 scFvs (5 μm) for 6 h at 37 °C. The cells were then permeabilized (or not) before staining. After immunofluorescence staining with rabbit IgG and FITC-labeled anti-rabbit IgG, the amount of protein taken up by the cells (permeabilized cells) or bound to the cell surface (non-permeabilized cells) was quantified by flow cytometry (*A* and *B*). Data are representative of three independent experiments. Confocal fluorescence images show 3D8 scFvs internalized within the permeabilized cells (*C*). Data are representative of four independent experiments. The cell nuclei were stained with Hoechst 33342 (*blue*). *Scale bar* = 10 μm.

We next analyzed uptake of the different scFvs in permeabilized HeLa cells and the amount of scFv bound to the surface of non-permeabilized cells by flow cytometry. Cells were incubated with the scFvs for 6 h at 37 °C. The cell membranes were then fixed and either permeabilized or not to distinguish cell surface-bound (Fig. 3, *A* and *B*, *left panels*) from endocytosed scFv (Fig. 3, *A* and *B*, *right panels*). Compared with wild-type 3d8 scFv, the three 3D8-Hi variants were internalized much less efficiently, and large amounts of 3D8-H2i and 3D8-H3i proteins remained bound to cell surface after washing (Fig. 3*A*). For the 3D8-Li variants, 3D8-L3i was endocytosed to the same extent as the wild-type 3D8 scFv, whereas 3D8-L1i and 3D8-L2i neither bound to nor entered the cells (Fig. 3*B*). The negative control, HW6 scFv, did not generate a signal. The flow cytometry data with permeabilized cells were supported by confocal microscopy (Fig. 3*C*). Thus, the cell-penetrating activity of 3D8 scFv was relatively resistant to the deactivation of LCDR3 compared with deactivation of the other CDRs.

**FIGURE 4.**
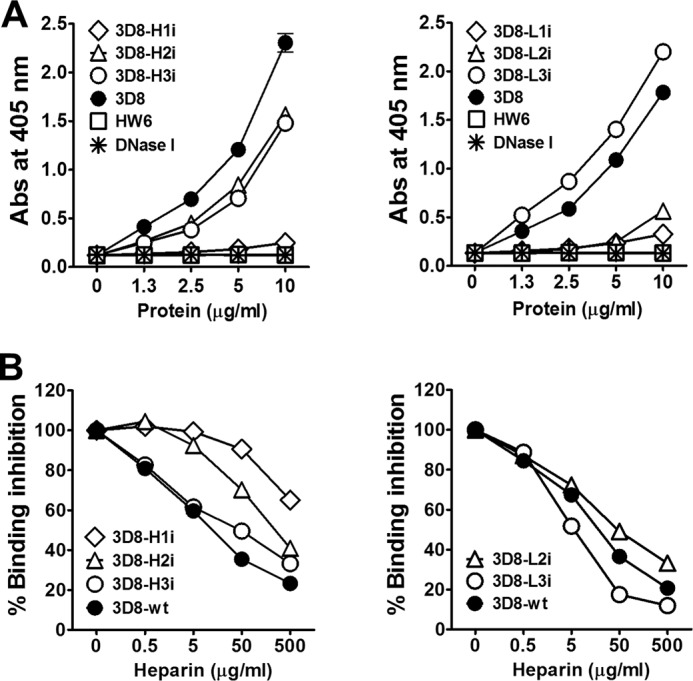
**Heparin binding activity of the 3D8 scFv variants.** *A*, ELISA for heparin binding activity of scFvs. Wells coated with heparin (10 μg/ml) were incubated with various concentrations of scFvs. *B*, competitive ELISA. Wells coated with bio-ss-(dN)_40_ DNA (1 μm) were incubated with scFvs (2.5 μg/ml) and heparin (varying concentrations). *A* and *B*, the scFvs bound to DNA were detected using rabbit IgG (1 μg/ml) followed by alkaline phosphatase-conjugated goat anti-rabbit IgG. Data represent the mean ± S.D. of sextuplicate wells from two independent experiments.

Previous studies suggest that heparan sulfate proteoglycans function as cell surface receptors that recognize anti-DNA Abs, and that anti-DNA Abs cross-react with negatively charged heparan sulfate epitopes (7, 24–26). Therefore, we next examined the heparin binding activity of the 3D8 variants. We found that 3D8-H2i, 3D8-H3i, and 3D8-L3i bound strongly to heparin, a soluble heparan sulfate analog, in a manner comparable with that of the wild-type scFv, whereas the negative controls (HW6 scFv and DNase I) did not bind to heparin (Fig. 4*A*). 3D8-H2i, 3D8-H3i, and 3D8-L3i were also shown to be DNA binders (Table 1). Moreover, DNA binding of the strong heparin binders (3D8-H2i, 3D8-H3i, 3D8-L3i, and wild-type 3D8 scFv) was inhibited by heparin in a concentration-dependent manner (Fig. 4*B*), indicating that heparan sulfate competes for the DNA-binding site of these scFvs. Taken together, the heparin binding activity of the scFvs correlated to either their cell surface binding (for 3D8-H2i and 3D8-H3i) or cell-penetrating (for 3D8-L3i and wild-type 3D8 scFv) activities, although cell surface binding was not necessarily correlated with cellular internalization.

##### DNA-hydrolyzing Activity of the scFvs

**FIGURE 5.**
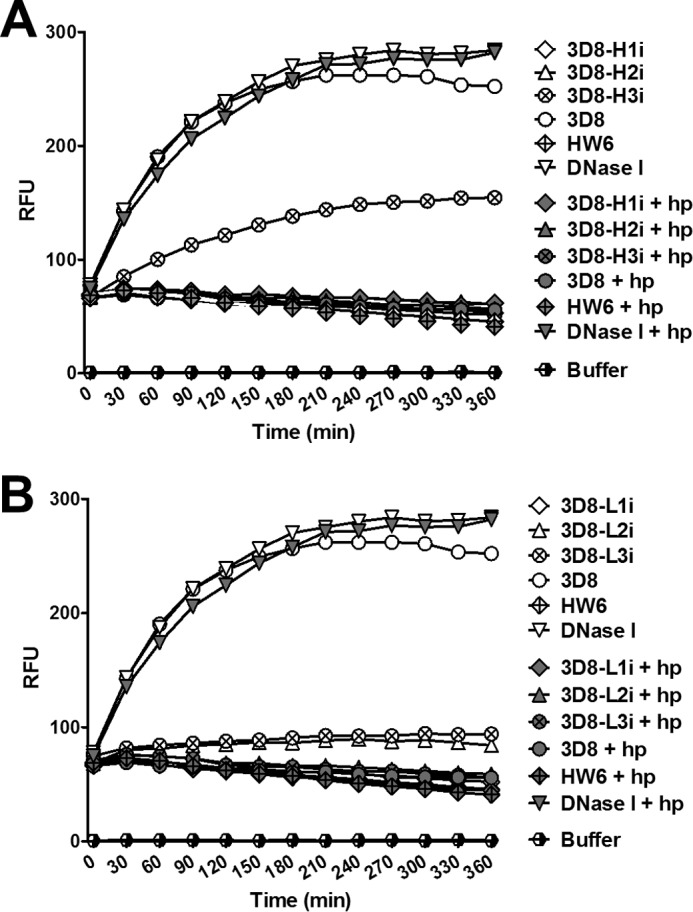
**FRET-based DNA cleavage assay.** ScFv (1 μm) or a mixture of scFv (1 μm) and heparin (10 μg/ml) were incubated with a double-labeled DNA substrate (250 nm) as described under “Experimental Procedures.” Fluorescence intensity was measured at 5-min intervals over 6 h in real time. *RFU*, relative fluorescence units; *hp*, heparin. Data are representative of four independent experiments.

The DNA-hydrolyzing activity of the different scFv constructs was analyzed using a FRET-based DNA cleavage assay in which the hydrolysis of a DNA substrate double-labeled with a fluorophore at the 5′ terminus and its quencher at the 3′ terminus was measured according to the increase in fluorescence intensity. The assay was performed in the presence or absence of heparin, which competes with the 3D8 scFvs for DNA-binding sites (Fig. 4*B*). We hypothesized that if the DNA-hydrolyzing activity of the 3D8 scFvs was inhibited by heparin, then this would rule out any false positives caused by contamination. The DNA-hydrolyzing activity of wild-type 3D8 scFv was completely blocked by heparin; however, the DNA-hydrolyzing activity of DNase I, which does not bind to heparin, was not affected by heparin as expected (Fig. 5). At least three scFv variants, 3D8-H3i (Fig. 5*A*), 3D8-L2i, and 3D8-L3i (Fig. 5*B*), retained DNA-hydrolyzing activity in the absence of heparin, although the activity of 3D8-H3i was reduced by 45% and that of 3D8-L2i and 3D8-L3i was reduced by 75%. None of the 3D8 variants showed DNA-hydrolyzing activity in the presence of heparin, confirming that the DNA-hydrolyzing activity of scFv is due to the 3D8 proteins rather than to any contaminating proteins.

##### Properties of CDR-derived Peptides

We analyzed the DNA binding/hydrolyzing, heparin binding, and cell-penetrating activities of CDR-derived peptides that were N-terminally labeled with either biotin or FITC. The amino acid sequence, length, and pI value of the six peptides corresponding to the CDRs of 3D8 scFv are shown in Table 2. Also shown are two control peptides: a positive control pep-Tat, which has a high cell penetration efficiency (28), and a negative control pep-VHFR3 derived from a part of the FR (framework region) in the 3D8 heavy chain.

**TABLE 2:** **The 3D8 CDR peptides and control peptides used in this study**

Peptide	Amino acid sequence	Length	pI
pep-H1	SYVMH	5	6.5
pep-H2	YINPYNDGTK	10	5.8
pep-H3	GAYKRGYAMDY	11	8.4
pep-L1	KSSQSLFNSRTRKNYLA	17	11.1
pep-L2	WASTRES	7	6.0
pep-L3	KQSYYHMYT	9	8.4
pep-VHFR3	KSSSTAYMEL	10	6.0
pep-Tat	GRKKRRQRRRPPQ	13	12.7

**FIGURE 6.**
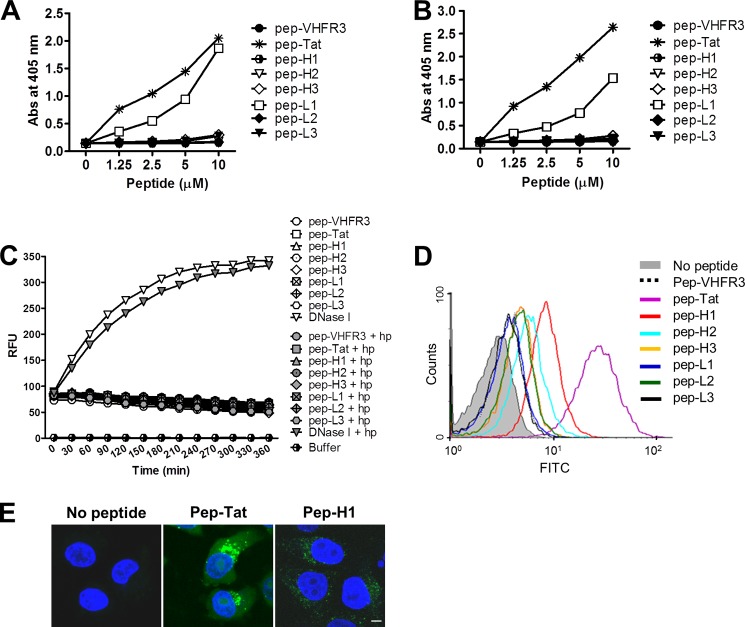
**Biochemical properties of 3D8 CDR-derived peptides.** *A* and *B*, ELISA for DNA and heparin binding activity. Wells coated with 10 μg/ml plasmid DNA (*A*) or 1 μg/ml heparin (*B*) were incubated with biotin-labeled peptides. The bound peptides were detected using alkaline phosphatase-conjugated streptavidin. Data represent the mean ± S.D. of triplicate wells and are representative of two independent experiments. *C*, FRET-based DNA cleavage assay. scFvs (1 μm) or a mixture of scFv (1 μm) and heparin (10 μg/ml) were incubated with a double-labeled single-stranded DNA substrate (500 nm) as described under “Experimental Procedures.” The fluorescence intensity was then measured in real time over 6 h (at 5-min intervals). *RFU*, relative fluorescence units. Data are representative of three independent experiments. *D* and *E*, cell-penetrating activity of the peptides. HeLa cells were incubated with FITC-labeled peptides (5 μm) for 6 h at 37 °C and then analyzed by flow cytometry (*D*) and confocal microscopy (*E*). Nuclei were stained with Hoechst 33342 (*blue*). *Scale bar* = 10 μm. Data are representative of three independent experiments.

ELISA results showed that the pI value of the peptide was directly proportional to its DNA binding (Fig. 6*A*) and heparin binding (Fig. 6*B*) activity. The degree of binding activity was pep-Tat (pI 12.7) > LCDR1 peptide (pep-L1; pI 11.1). Thus, electrostatic interactions between the basic residues within the proteins and the negatively charged DNA and heparin molecules may be the primary factor that determines the strength of binding. None of the CDR-derived peptides showed DNA-hydrolyzing activity (Fig. 6*C*). Only the pep-H1 and pep-H2 peptides showed cell-penetration activity, but the level of entry was <10% that observed for the Tat control peptide (Fig. 6*D*). A representative confocal microscopic image of the internalized pep-H1 is shown in Fig. 6*E*. These data indicate that the amino acid sequence of a CDR-derived peptide, rather than its pI, determines whether it is internalized by a cell. We expected pep-L1 to penetrate the cells because LCDR1-deactivated scFv failed to show either DNA binding or cell penetration activity; however, pep-L1 did not penetrate the cells. This suggests that soluble pep-L1 may have a bioactive behavior different from that when it is incorporated between the FRs.

##### Properties of the Hybrid Ab, HW6/3D8L1

**FIGURE 7.**
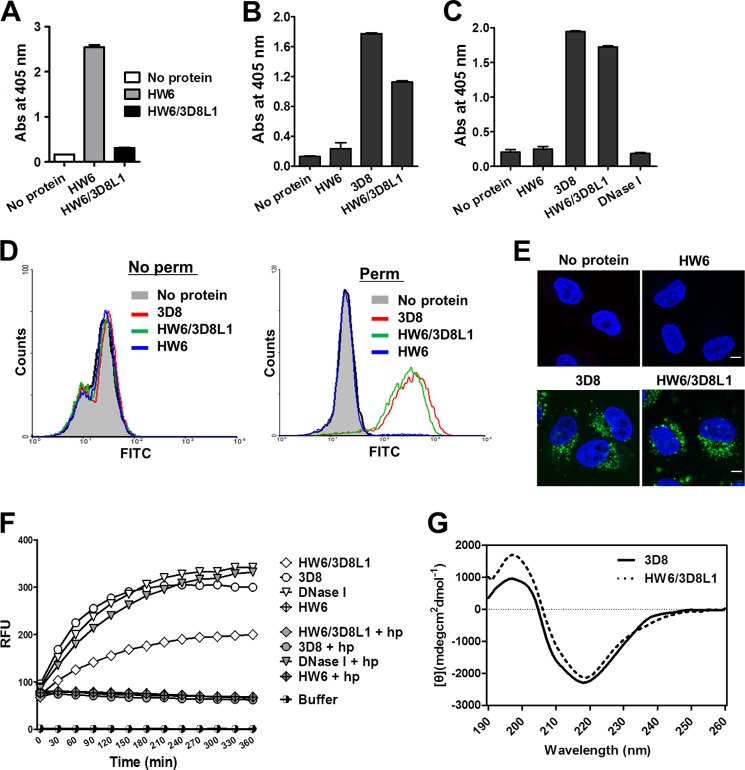
**Biochemical properties of HW6/3D8L1.** *A–C*, ELISA of the binding of scFvs to DR5, DNA, and heparin. scFvs were incubated in wells coated with 5 μg/ml DR5 protein (*A*), 1 μm bio-ss-(dN)_40_ DNA (*B*), or 1 μg/ml of heparin (*C*). The bound proteins were detected using rabbit IgG followed by alkaline phosphatase-conjugated anti-rabbit IgG. Data represent the mean ± S.D. of triplicate wells from two independent experiments. *D* and *E*, cell-penetrating activity of scFvs. HeLa cells were incubated with scFvs (5 μm) for 6 h at 37 °C and then either permeabilized or not. The cells were then incubated with rabbit IgG followed by FITC-labeled anti-rabbit IgG and then analyzed by flow cytometry (*D*, *right panel*, permeabilized cells; *left panel*, non-permeabilized cells) and confocal microscopy (*E*: permeabilized cells). Cell nuclei were stained with Hoechst 33342 (*blue*). *Scale bar* = 10 μm. Data are representative of three independent experiments. *F*, FRET-based DNA cleavage assay. ScFvs (1 μm) or a mixture of scFvs (1 μm) and heparin (10 μg/ml) were incubated with a double-labeled DNA substrate (250 nm) as described under “Experimental Procedures.” The fluorescence intensity was then measured in real time over 6 h (at 5-min intervals). *RFU*, relative fluorescence units. Data are representative of four independent experiments. *G*, spectra of HW6/3D8L1 obtained with far-UV CD spectroscopy.

To further identify the role of 3D8-LCDR1, we generated a hybrid Ab HW6/3D8L1 in which the LCDR1 of HW6 scFv (human anti-DR5 (death receptor 5)) was replaced with the LCDR1 from 3D8 (KSSQSLFNSRTRKNYLA: 17 amino acids). HW6/3D8L1 did not retain DR5 binding activity (Fig. 7*A*) but acquired the DNA binding (Fig. 7*B*), heparin binding (Fig. 7*C*), cell-penetrating (Fig. 7, *D* and *E*), and DNA-hydrolyzing (Fig. 7*F*) activities of the wild-type 3D8 scFv, although DNA-hydrolyzing activity was reduced by ∼50% compared with wild-type 3D8 scFv. The CD spectra of HW6/3D8L1 showed a predominantly β-sheet secondary structure, similar to wild-type 3D8 (Fig. 7*G*). Taken together, the findings suggest that HW6/3D8L1 folds properly and exhibits properties similar to that of wild-type 3D8 scFv. Considering the unremarkable features of LCDR1 shown in Fig. 5, it appears that the LCDR1 of 3D8 is fully functional only when incorporated into an appropriate protein scaffold.

## DISCUSSION

The present study investigated for the first time the functional consequences of deactivating the CDRs of Abs that have multiple activities, such as 3D8 scFv. Interestingly, the activities of the CDR-deactivated scFv variants were affected differently (unaffected or only partially affected), depending on which CDR was deactivated, and deactivation of LCDR1 abolished all activities. The DNA binding activity of 3D8 scFv was relatively resistant to the deactivation of single CDRs. This observation was unexpected considering the properties of mouse anti-DNA mAb 9D7, which is also able to penetrate cells; a single amino acid change (from Arg to Ala) within either the HCDR2 or HCDR3 region significantly decreased both its DNA binding and cell-penetration activities (7).

Our previous study revealed the importance of His residues within HCDR1 and LCDR3 of 3D8 scFv for DNA hydrolysis (15). However, substituting Ala residues for His within HCDR1 and LCDR3 did not affect its DNA binding capacity.^3^ This demonstrates that DNA binding and DNA hydrolysis can be separately manipulated by changing critical portions of the CDR. Our results showed that the DNA binding, DNA-hydrolyzing, and cell-penetrating activities of 3D8 scFv can be selectively manipulated by deactivating different CDRs.

Few studies have been conducted on the functional consequences of single complete CDR-scaled changes in Ab. This may be due to the commonly held view that the properties of an Ab are dependent upon the combined function of all six CDR loops; therefore, deactivation of a single complete CDR loop would be expected to severely impair Ab activity and structure. Furthermore, substitutions or insertions of amino acids within the CDRs have a marked impact on Ab affinity (7, 29–31) and aggregation (32). However, contrary to this concern, we observed that the (Gly/Ser)*_n_* grafted into the loop structures of each CDR are stabilized by the β-sheet FRs of the variable domains of 3D8 Ab (Fig. 2). All scFv variants generated for this study were amply obtained (2–5 mg/liter) from the supernatant of bacterial culture in a soluble form, not as intracellular inclusion bodies formed by the aggregation of misfolded proteins. Therefore, it appears that 3D8 scFv can structurally accommodate single CDR deactivation. However, although CDR deactivation worked well for the identification of functional CDRs in the multifunctional mAb 3D8 scFv, this strategy may not be applicable to the majority of mAbs. Studies with a variety of multifunctional mAbs will be needed to know if this strategy can be applied to other mAbs.

We reported previously that the _H3_Tat-3D8 Ab, in which HCDR3 of 3D8 is replaced with a basic Tat peptide, acquired properties associated with the Tat peptide with no distortion of the intrinsic activities of the parent 3D8 Ab (10). Our current finding is consistent with the pliability of HCDR3 of 3D8 in that deactivation of HCDR3 affected DNA binding/hydrolysis and the cell-penetrating activity of 3D8 scFv to a much lesser extent than that of the other CDRs (Table 1 and Figs. 3 and 5). Functional peptides generated from non-Ab proteins can be grafted into the CDR loops of Ab scaffolds to create functionalized Abs with a similar or higher affinity for antigens and a longer half-life than the original peptides (26–28). HCDR3 of 3D8 may be a potential target region into which a functional peptide can be grafted to create a newly functionalized Ab protein.

The LCDR1 peptide (17 amino acids) of 3D8, which is not flanked by FRs and may lack a stable tertiary structure, showed no DNA hydrolysis or cell-penetration activity except for low levels of DNA and heparin binding (Fig. 6). This is in contrast to reports showing that the CDR2-CDR3 peptide of the cell-penetrating anti-DNA mAb F4.1 can penetrate cells (10) and that CDR-derived peptides could function in a manner similar to that of the original Ab (10, 18).

Our conclusion that LCDR1 of 3D8 is the most important domain in terms of activity is supported by the observations that (1) deactivation of LCDR1 abolished all 3D8 activities, and that (2) surprisingly, grafting LCDR1 of 3D8 into an appropriate protein scaffold, such as HW6 scFv, was sufficient to confer DNA binding and cell-penetrating activities similar to those of the parent 3D8 scFv as well as a significant amount (50%) of DNA-hydrolyzing activity (Fig. 7). Interestingly, the DNA-hydrolyzing activity of HW6/3D8L1 was comparable to that of humanized 3D8 scFv, in which all six CDRs of 3D8 were grafted into corresponding human FRs (data not shown).

The function of the 3D8 LCDR1 domain might only be expressed when it is grafted into mAbs that possess adequate FRs, as in the case of mAb HW6. Actually, when we transferred the LCDR1 domain of 3D8 scFv into a chicken scFv mAb that is specific for the sporozoite antigen of the parasite *Eimeria tenella*, the hybrid Ab did not exhibit any of the activities of 3D8 scFv (data not shown). A full understanding of the role of the 3D8 LCDR1 domain in mAb function could be obtained by investigating whether the transfer of this domain into a wide range of mAbs results in their functionalization. In the protein engineering field, functional CDRs derived from Abs have been grafted into non-Ab scaffold proteins such as GFP, neocarzinostatin, and human fibronectin to create functionalized proteins (33–35). It may be worthwhile grafting the 3D8 LCDR1 domain onto these non-Ab scaffolds.

In conclusion, the present study showed that the deactivation of single CDRs in 3D8 scFv resulted in 3D8 scFvs with different properties and revealed an important role for LCDR1 in all 3D8 scFv activities (DNA binding, DNA hydrolysis, and cell penetration). This specific, whole CDR-deactivating strategy, rather than the commonly employed point-mutational strategy, might provide insight into the molecular properties of multifunctional Abs and allow the identification of functional CDRs in a variety of Abs.

## Footnotes

3 J. Lee, H.-J. Kim, J. Roh,Y. Seo, M. Kim, H.-R. Jun, C. D. Pham, and M.-H. Kwon, unpublished data.

## Footnotes

2 The abbreviations used are:

CDR: complementarity-determining region
scFv: single chain variable fragment
FR: framework region
SPR: surface plasmon resonance
HCDR: heavy chain
LCDR: light chain
Ab: antibody.
